# Imaging the human placental microcirculation with micro-focus computed tomography: Optimisation of tissue preparation and image acquisition

**DOI:** 10.1016/j.placenta.2017.09.013

**Published:** 2017-12

**Authors:** Rosalind Pratt, J. Ciaran Hutchinson, Andrew Melbourne, Maria A. Zuluaga, Alex Virasami, Tom Vercauteren, Sebastien Ourselin, Neil J. Sebire, Owen J. Arthurs, Anna L. David

**Affiliations:** a Translational Imaging Group, Dept. Medical Physics and Biomedical Engineering, University College London, Malet Place Engineering Building, Gower Street, London WC1E 6BT, UK; b Institute for Women's Health, University College London, 86-96 Chenies Mews, London WC1E 6HX, UK; c University College London Great Ormond Street Institute of Child Health, University College London, 30 Guilford Street, London, WC1N 1EH, UK; d Department of Histopathology, Great Ormond Street Hospital for Children NHS Trust, Great Ormond Street, London, WC1N 3JH London, UK; e Wellcome / EPSRC Centre for Surgical and Interventional Sciences, University College London, London, UK; f Paediatric Radiology, Great Ormond Street Hospital for Children NHS Trust, Great Ormond Street, London, WC1N 3JH London, UK

**Keywords:** Micro-CT, Optimisation, Perfusion, Contrast

## Abstract

Micro-CT provides 3D volume imaging with spatial resolution at the micrometre scale. We investigated the optimal human placenta tissue preparation (contrast agent, perfusion pressure, perfusion location and perfusion vessel) and imaging (energy, target material, exposure time and frames) parameters.

Microfil (Flow Tech, Carver, MA) produced better fill than Barium sulphate (84.1%(±11.5%)vs70.4%(±18.02%) p = 0.01). Perfusion via umbilical artery produced better fill than via chorionic vessels (83.8%(±17.7%)vs78.0%(±21.9%), p < 0.05), or via umbilical vein (83.8%(±16.4%)vs69.8%(±20.3%), p < 0.01). Imaging at 50 keV with a molybdenum target produced the best contrast to noise ratio. We propose this method to enable quantification and comparison of the human fetoplacental vascular tree.

## Highlights

• μCT generates high resolution quantifiable placental vascular imaging.
• We present an optimised perfusion technique for human placental μCT imaging.
• We present optimised μCT parameters for human placental angiography.

## 1 Introduction

Fetal health and development is intricately bound with human placental circulation, yet there is no validated quantitative method with which to assess vascularisation of the human placenta. Developing a quantitative method may improve our ability to investigate, and therefore understand, normal placental function and pathologies such as fetal growth restriction, stillbirth and twin-to-twin transfusion syndrome.

Micro-focus Computed Tomography (micro-CT) provides three-dimensional volume imaging with spatial resolution at the micrometre scale. The technique has been used to investigate the branching structure and tortuosity of the fetoplacental circulation of mouse placentae [1] [2], and shown differences in vascular density of the human placenta between normally grown and growth restricted pregnancies [3], [4].

This study was designed to develop optimised tissue-specific preparation and micro-CT imaging parameters, in order to provide a validated approach to human placenta micro-CT.

## 2 Method

This series of experiments is divided into two sections; investigating tissue preparation techniques, and then micro-CT imaging parameters. The full experimental methodology is described in supplementary data.

### 2.1 Tissue acquisition

Experimental procedures were approved by Bloomsbury National Research Ethics Service Committee and by University College London Hospital Research and Development (REC Reference number 133888). Placentas delivered by elective term caesarean section were taken directly to the laboratory, had the membranes trimmed, and the amnion removed.

### 2.2 Tissue preparation comparators

We designed experiments to compare (Table 1):

• Contrast agent – comparing Barium sulphate with Microfil (Flow Tech, Carver, MA.).
• Perfusion pressure – comparing manual pressure with no quantification of perfusion pressure, with controlled pressure of 60  mmHg, physiologically relevant to fetal life [5], [6], [7].
• Cannulation location – comparing perfusion via the umbilical artery with perfusion via a chorionic artery.
• Arterial or Venous Cannulation – comparing perfusion via cannulation of the umbilical artery with perfusion via the umbilical vein.

**Table 1:** Comparison of placental tissue preparation and micro-CT imaging parameters used in this study and in two previous studies, and optimised protocol as determined by the results of this study. SNR = signal to noise ratio.

	Langheinrich [4] (Human)	Rennie et al.[10] (Mouse)	Assessment Parameters	Optimised Protocol
Tissue Preparation				
Contrast Agent	Microfil and BaSO4 in gelatin	Microfil	Microfil and BaSO4 in gelatin	Microfil
Perfusion Pressure (mmHg)	74	Not reported	Manual pressure and 60	No difference Manual and 60 mmHg give equivalent results
Perfusion Location	Chorionic (peripheral) perfusion	Umbilical (central) Perfusion	Chorionic (peripheral) and umbilical (central) perfusion	Central vessel, ideally umbilical vessel
Perfusion Vessel	Chorionic plate artery	Umbilical Artery/Umbilical Vein	Chorionic artery/Umbilical artery/Umbilical vein	Artery
Tissue sampling technique	8 × 2 mm full thickness blocks	Whole placenta	8 × 2 cm full thickness blocks	Dependent on magnification and field of view required
Micro CT Imaging				
Cone-beam energy (keV)	60	80	30-100 in 10 keV increments	50
Target material	Not reported	Not reported	Tungsten, Molybdenum, Copper	Molybdenum
Isotropic voxel size (μm)	13 and 4	13	13	Dependent on magnification and field of view required
Radiograph exposure time (ms)	2400	Not reported	500/1000	Balance with throughput 1000 gives good SNR
Number of projections	400	720	3141/6282/12564	Balance with throughput 3141 gives good SNR

The fetal vessel of interest was cannulated, and a cut made in the main draining vessel close to the point of cannulation, to create a fluid exit vent. 0.9% sodium chloride solution with 5IU heparin/ml was perfused until the outflow ran clear, then contrast agent was perfused until the chorionic vasculature was fully perfused and contrast agent was seen in the draining vessel. The vessel was occluded and the contrast agent was left to set. The placenta was dissected into 2 × 2cm full thickness blocks, which were fixed in 4% formalin for a minimum of 48 h. One full thickness section stained with hematoxylin and eosin (H&E) was cut for every block and 6 micrographs at x100 magnification taken (see supplementary material).

Histological analysis was done in FIJI (ImageJ Version 2.0.0-rc-54/1.51f) [8]. Vascular fill was calculated for each micrograph as shown in equation one.

(1) $$Vascular\;Fill\;\left(\%\right)=\left(\frac{Total\;Perfused\;Vessel\;Area}{Total\;Perfused\;Vessel\;Area+Total\;Unperfused\;Vessel\;Area}\right)\times 100$$

### 2.3 Micro-CT imaging comparators

We designed experiments to compare (Table 1).

• Energy level – from 30 to 100 keV in 10 keV increments.
• Target material – comparing Tungsten, Copper and Molybdenum.
• Exposure time–500 and 1000 ms
• Averaged frames per projection–1 and 2

A 2 × 2cm full thickness block of human placenta was repeatedly imaged (XT H 225 ST Micro-CT, Nikon Metrology, Tring, UK) adjacent to a 3 mm internal diameter tube filled with Microfil. Scans were reconstructed using a modified Feldkamp filtered back projection algorithm with proprietary software (CTPro3D; Nikon Meterology), and the average greyscale values of recorded areas of interest drawn over placenta, Microfil and air were calculated. The contrast to noise ratio was calculated as shown in equation two.

(2) $$Contrast\;to\;Noise\;ratio\;\left(CNR\right)=\;\frac{\left(Placenta\;Grey\;Scale\;Value-Microfil\;Grey\;Scale\;Value\right)}{Standard\;Deviation\;of\;Signal\;of\;Air}$$

### 2.4 Statistical analysis

Data is presented as mean ± SD. Statistical analysis was done in SPSS Statistics (IBM version 23). Group comparison was performed using independent sample t-tests with significance set at 95%.

## 3 Results

### 3.1 Tissue preparation comparators

There was lower mean vascular fill with barium sulphate than Microfil (70.4% (±18.02%) BaSo4 vs 84.1% (±11.5%) Microfil, (p = 0.01)) and barium sulphate was seen in the extravascular space in all three blocks sampled (47% of micrographs), whereas Microfil was never seen in the extravascular space.

There was no significant difference in vascular fill between manual or controlled 60  mmHg perfusion pressure (77.8%(±13.9%) manual vs 78.0%(±21.9%) controlled pressure p = 0.95). Perfusion via an umbilical artery achieved higher vascular fill than perfusion via a more peripheral chorionic vessel (83.8%(±17.7%) umbilical artery vs 78.0%(±21.9%) chorionic artery, p < 0.05). Umbilical arterial perfusion produced higher vascular fill than umbilical venous perfusion (83.8%(±16.4%) umbilical artery vs 69.8%(±20.3%) umbilical vein p < 0.01) (see Table 1 for summary, and supplementary data Table 1 for full results).

### 3.2 Micro-CT imaging parameters

**Fig. 1:**
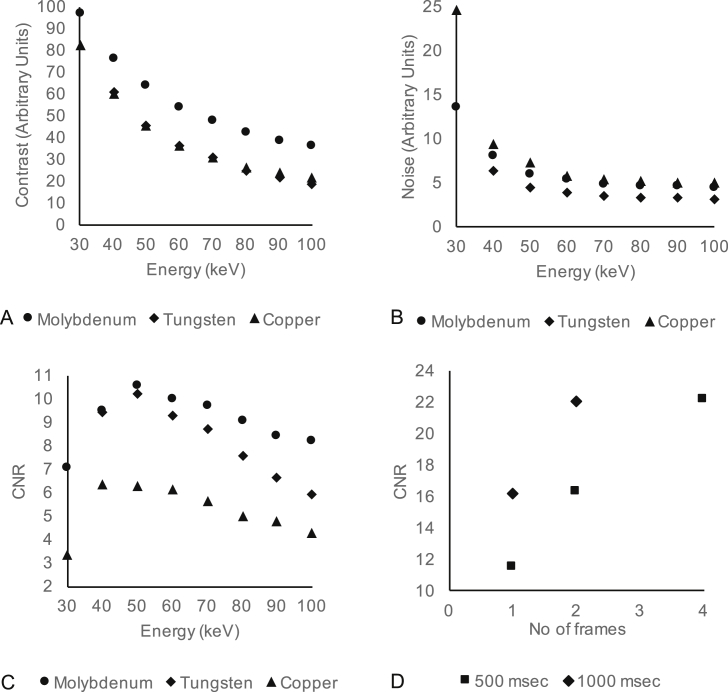
Identifying optimal micro-CT imaging parameters for Contrast to Noise Ratio (CNR). **A:** Contrast (defined as Microfil Grey Scale value – placental issue Grey Scale value, arbitrary units) between placenta and Microfil grey scale value with increasing energy for Molybdenum, Tungsten and Copper target. **B:** Standard deviation of the signal in air, the image noise, with increasing energy for Molybdenum, Tungsten and Copper target. **C:** Contrast to noise ratio with increasing energy for Molybdenum, Tungsten and Copper target. **D:** Effect of increasing the exposure time and the averaged frames per projection on the CNR.

Contrast and noise were both greatest at the lower energy levels (Fig. 1A/B). The optimal CNR was with Molybdenum target at 50 keV (Fig. 1C). Increasing exposure time from 500 ms to 1000 ms and averaged frames per projection reduced the noise and improved the CNR (Fig. 1D) at the cost of imaging time and throughput (Table 1).

## 4 Discussion

We have established optimal tissue and imaging parameters for placental angiographic micro-CT (Table 1). Our studies show that Microfil is a superior contrast agent to barium suphate, and that central and arterial perfusion are superior to peritheral and venous perfusion. Contrast to noise ratio is optimal when imaging with 50 keV energy, with a Molybdenum target. Increasing the number of projection and exposure time improves CNR at the cost of throughput. Our studies found 1000 ms exposure time and 3141 projections over 360° rotation produced good CNR with a 54 min scan time.

This approach can be used to investigate the microcirculation of the human placenta. The technique benefits from its high resolution and large field of view, allowing images of the vascular tree to be captured from the chorionic plate to the intermediate villous vessels (see supplementary data for images).

Micro-CT allows measurement of vascular density and analysis of the structure of the vascular trees, which could improve our understanding of the heterogeneity within normal placentae, and the structural changes associated with diseases such as early and late intrauterine growth restriction.

## Funding

ALD and SO are supported at UCLH/UCL by funding from the Department of Health NIHR Biomedical Research Centre's funding scheme. OJA is funded by a National Institute for Health Research (NIHR) Clinician Scientist Fellowship (NIHR-CS-012-002), NJS is funded by an NIHR Senior Investigator award, Great Ormond Street Children’s Charity and the Great Ormond Street Hospital NIHR Biomedical Research Centre [ORMBRC-2012-1].

This paper presents independent research funded by the National Institute for Health Research (NIHR). The views expressed are those of the author(s) and not necessarily those of the NHS, the NIHR or the Department of Health.

## Statements of contribution

Rosalind Pratt

I declare that I have contributed to the design, acquired the data and performed the analysis of this study, that I am the primary contributor to the manuscript, and that I have seen and approved the final version. I have no conflicts of interest to declare.

J. Ciaran Hutchinson

I declare that I have contributed to the design, execution and analysis of this study and that I have seen and approved the final version. I have no conflicts of interest to declare.

Andrew Melbourne

I declare that I have contributed to the design, execution and analysis of this study and that I have seen and approved the final version. I have no conflicts of interest to declare.

Maria Zuluaga Valencia

I declare that I have contributed to the automated FIJI analysis of histology, and that I have seen and approved the final version. I have no conflicts of interest to declare.

Alex Virasami

I declare that I have contributed to histological analysis in this study and that I have seen and approved the final version. I have no conflicts of interest to declare.

Tom Vercauteren

I declare that I have contributed to the design, execution and analysis of this study and that I have seen and approved the final version. I have no conflicts of interest to declare.

Sebastien Ourselin

I declare that I have contributed to the design, execution and analysis of this study and that I have seen and approved the final version. I have no conflicts of interest to declare.

Neil Sebire

I declare that I have contributed to the design, execution and analysis of this study and that I have seen and approved the final version. I have no conflicts of interest to declare.

Owen J Arthurs

I declare that I have contributed to the design, execution and analysis of this study and that I have seen and approved the final version. I have no conflicts of interest to declare.

Anna L David

I declare that I have contributed to the design, execution and analysis of this study and that I have seen and approved the final version. I have no conflicts of interest to declare.

## Conflicts of interest

None.
