# Supplementary material for: Imaging the human placental microcirculation with micro-focus computed tomography: Optimisation of tissue preparation and image acquisition
Source: Placenta. 2017 Dec;60:36–9. doi: 10.1016/j.placenta.2017.09.013 (PMC5730539; doi:10.1016/j.placenta.2017.09.013)
Supplement: Supplementary data 1 [file mmc1.docx]

| Perfusion Technique | Number of placentas perfused | Number of Placental Blocks | Number of Micrographs | Number of Micrographs excluded (%) | Number of vessels counted | Mean Vascular fill % (+/-SD) | P value |
| --- | --- | --- | --- | --- | --- | --- | --- |
| Contrast Agent  (Chorionic artery cannulation, manual perfusion pressure) | | | | | | | |
| BaSO4 | 1 | 3 | 18 | 0 (0) | NA | 70.4 (18.02) | 0.01* |
| Microfil |  | 3 | 18 | 0 (0) | NA | 84.1 (11.5) |  |
| Perfusion Pressure  (Microfil as contrast agent, chorionic artery cannulation) | | | | | | | |
| Manual Pressure | 4 | 13 | 78 | 18 (23) | 15624 | 77.7 (13.9) | 0.95 |
| Controlled 60mmHg Pressure | 4^^^ | 14 | 84 | 12 (14) | 14332 | 78.0 (21.9) |  |
| Cannulation Location  (Microfil as contrast agent, controlled perfusion pressure) | | | | | | | |
| Umbilical Artery | 4^#^ | 27 | 186 | 36 (19) | 17263 | 83.9 (15.85) | 0.04* |
| Chorionic Artery | 4^^^ | 14 | 84 | 12 (14) | 14332 | 78.0 (21.9) |  |
| Arterial or Venous Cannulation  (Microfil as contrast agent, controlled perfusion pressure) | | | | | | | |
| Umbilical artery | 4^#^ | 27 | 186 | 36 (19) | 17263 | 83.9 (15.85) | <0.01* |
| Umbilical vein | 2 | 8 | 48 | 0 (0) | 15503 | 69.8 (20.3) |  |

Supplementary Data Table 1: Results from perfusion optimisation experiments. N = 15 placenta, superscripts symbols (^^,#^) show the same group of placentas used in different comparisons. P values are independent student t-test comparison of means, significance set at 0.05, denoted with *. NA = not available.
