# Supplementary material for: Imaging the human placental microcirculation with micro-focus computed tomography: Optimisation of tissue preparation and image acquisition
Source: Placenta. 2017 Dec;60:36–9. doi: 10.1016/j.placenta.2017.09.013 (PMC5730539; doi:10.1016/j.placenta.2017.09.013)
Supplement: Supplementary data 2 [file mmc2.pdf]

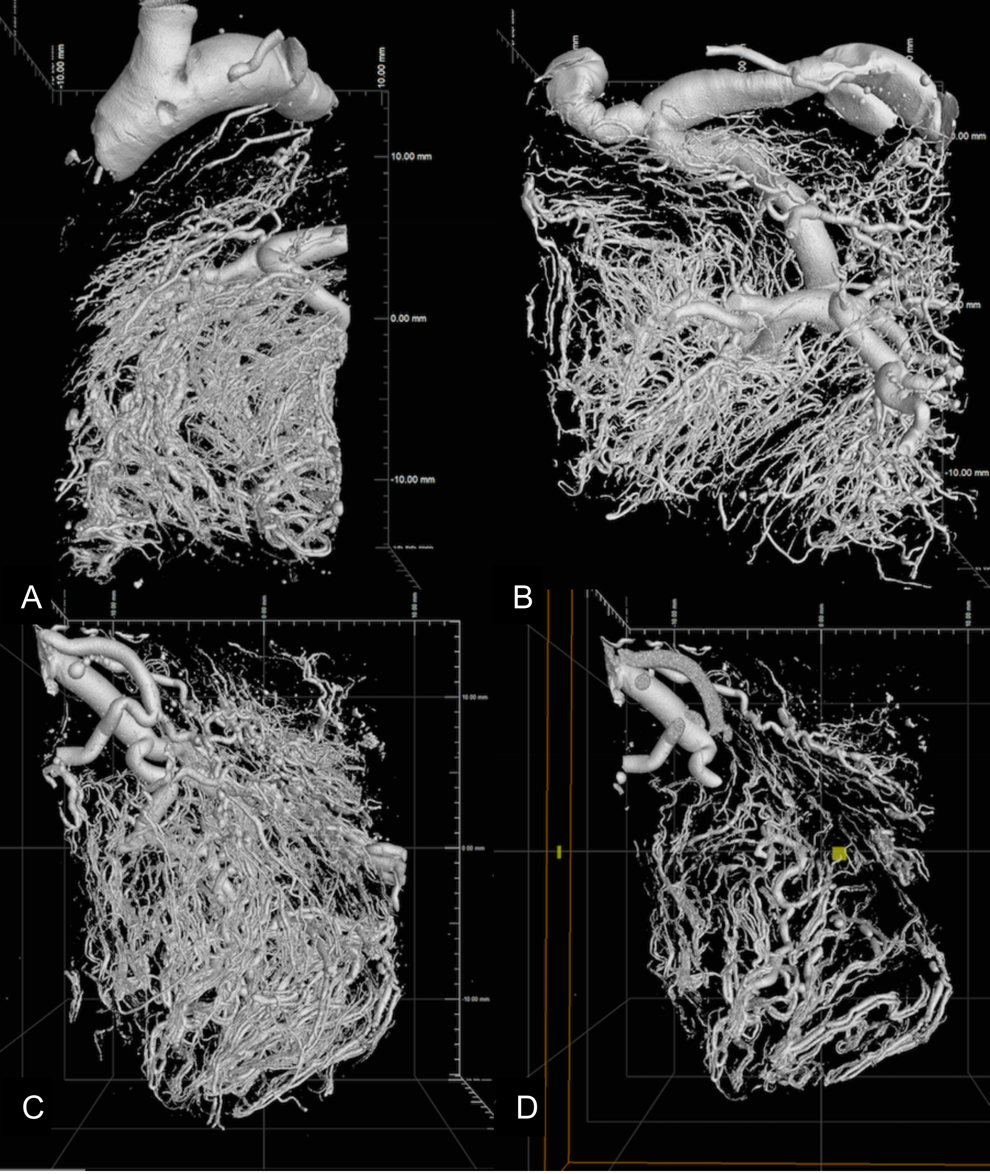

Micro-CT Images of the Human Feto-placental Circulation. Surface renderings made using VG Studio Max 2.2 (Volume Graphics GmbH, Heidelberg, Germany). A-C show three volume renderings of placental blocks from one placenta, and D a slice through volume C.
