# Supplementary material for: Imaging the human placental microcirculation with micro-focus computed tomography: Optimisation of tissue preparation and image acquisition
Source: Placenta. 2017 Dec;60:36–9. doi: 10.1016/j.placenta.2017.09.013 (PMC5730539; doi:10.1016/j.placenta.2017.09.013)
Supplement: Supplementary data 3 [file mmc3.docx]

Imaging the Human Placental Microcirculation with Micro-focus Computed Tomography: Optimisation of Tissue Preparation and Image Acquisition

Rosalind Pratt, J. Ciaran Hutchinson, Andrew Melbourne, Maria A. Zuluaga, Alex Virasami, Tom Vercauteren, Sebastien Ourselin, Neil J. Sebire, Owen J. Arthurs*, Anna L. David*.

Methodology for Optimisation of Tissue Imaging for Human MicroCT Experiments

Background

The aim of this experiment was to find a MicroCT imaging protocol that optimised the contrast to noise ratio for human placenta perfused with Microfil (Flow Tech, Carver, MA.). We hypothesised that lower energy would optimise contrast, but increase noise and reduce penetration. Increasing exposure time and frames per project would decrease noise, but this must be balanced with the imaging time and required throughput. The experiment was designed to find the balance of these factors that achieved optimal imaging.

Design

Experimental procedures were approved by Bloomsbury National Research Ethics Service Committee and by University College London Hospital Research and Development (REC Reference number 133888). Women undergoing elective caesarean section at term gave written consent for their placenta to be used.

Methodology

A 2x2cm full thickness block of human placenta was mounted for imaging next to a 3mm internal diameter tube of Microfil

This was imaged repeatedly using the XT H 225 ST Micro-CT, Nikon Metrology, Tring, UK using the following protocol:

- With a Tungsten target, changing the set energy level from 30–100 keV in 10 keV increments.
- With a Copper target, changing the set energy level from 30–100 keV in 10 keV increments.
- With a Molybdenum target, changing the set energy level from 30–100 keV in 10 keV increments.
  - Whilst maintaining exposure time at 500ms per projection, 3141 projections through 360° rotation, and adjusting the current to get adequate penetration, maintain brightness and avoid saturation.

The same placenta and Microfil model was then imaged with the Molybdenum target at 50keV enegery:

- Changing the exposure time from 500ms to 1000ms.
- Changing the averaged frames per projection from one to two.

The image volume was reconstructed using a modified Feldkamp filtered back projection algorithm with proprietary software (CTPro3D; Nikon Meterology). A circular area of interest with 1mm radius was drawn in the reconstructed volume over the same area of placenta, Microfil and air at slices 660 and 1320 of each 2000 slice volume (as background noise changes slightly with slice height) and the mean greyscale value and standard deviation for each was calculated and recorded. The contrast to noise ratio was calculated for each experiment with the averaged value as shown in equation two:

$$Contrast to Noise ratio \left( CNR \right)= \frac{\left( Placenta Grey Scale Value-Microfil Grey Scale Value \right)}{Standard Deviation of Signal of Air}$$

Equation 2
