# Supplementary material for: Imaging the human placental microcirculation with micro-focus computed tomography: Optimisation of tissue preparation and image acquisition
Source: Placenta. 2017 Dec;60:36–9. doi: 10.1016/j.placenta.2017.09.013 (PMC5730539; doi:10.1016/j.placenta.2017.09.013)
Supplement: Supplementary data 4 [file mmc4.docx]

Imaging the Human Placental Microcirculation with Micro-focus Computed Tomography: Optimisation of Tissue Preparation and Image Acquisition

Rosalind Pratt, J. Ciaran Hutchinson, Andrew Melbourne, Maria A. Zuluaga, Alex Virasami, Tom Vercauteren, Sebastien Ourselin, Neil J. Sebire, Owen J. Arthurs*, Anna L. David*.

Methodology for Optimisation of Tissue Preparation for Human Micro-CT Experiments

Background

The aim of this research was to compare four important tissue perfusion preparation parameters for human perfusion for MicroCT Imaging.

- Contrast agent – comparing Barium sulphate with Microfil (Flow Tech, Carver, MA.). We hypothesised that Microfil would more completely fill the microcirculation, as seen on histological analysis, than the barium sulphate preparation, as previously described[1], and that barium sulphate may leak into the extravascular space, whilst Microfil would remain intravascularly.
- Perfusion pressure – comparing manual pressure with no quantification of perfusion pressure, but whilst observing for vascular fill and evidence of vascular dilation with controlled pressure of 60mmHg, a value that is physiologically relevant to fetal life[2,3,4]. We hypothesised that controlled perfusion pressure would give a more even fill, allowing the tree to fill as similarly as it would in utero as possible.
- Cannulation location – comparing perfusion via the umbilical artery with perfusion via a chorionic artery. We hypothesised that perfusion through the central umbilical artery would give a more complete fill than perfusion of a peripheral chorionic vessel, as all feeding vessels would be perfused.
- Arterial or Venous Cannulation – comparing perfusion via cannulation of the umbilical artery with perfusion via the umbilical vein. We hypothesised that the more muscular arterial wall would be more elastic than the venous wall, maintaining perfusion pressure and giving a better fill of the microcirculation.

Design

Experimental procedures were approved by Bloomsbury National Research Ethics Service Committee and by University College London Hospital Research and Development (REC Reference number 133888).

Inclusion Criteria

- Women undergoing elective caesarean section after 38 completed weeks of pregnancy.
- No maternal complications of pregnancy, including but not limited to diabetes (gestational or pre-existing), hypertension, and pre-eclampsia.
- Birth weight above the 10^th^ centile, with no neonatal complications at delivery requiring resuscitation or admission to the Neonatal Unit.

Methodology

After delivery, the placenta was taken directly to the laboratory, the membranes trimmed and the amnion removed. The vessel of choice was cannulated using an 18-22 gauge cannula (depending on the size of vessel) and a small cut made in the main draining vessel close to the point of cannulation, to create a fluid exit vent. 0.9% sodium chloride solution with 5IU heparin/ml was perfused until the outflow ran clear to prevent vessel occlusion secondary to thrombosis. This required between 200-500ml heparinised saline, depending on the volume of tissue being perfused. The contrast agent of choice was perfused into the vasculature until all the vessels on the chorionic plate were seen to be filled, and some contrast agent was seen in the draining vessel. The cannulated vessel and fluid exit vent were occluded and the contrast agent left to set in accordance with manufacturer instructions.

Once set, the placenta was cut into 2cm wide strips through the perfused area of interest, and then cut these strips into 2cm blocks.

- For segment perfusion all blocks were taken for histological analysis (n=3-4).
- For whole placental perfusion blocks selected at random from different areas of the placenta and different distances from the cord insertion were taken (n=4-8 per placenta).

The blocks were fixed in 30ml 4% formalin at room temperature for a minimum of 48 hours.

Tissue Preparation Comparators

*Contrast agent*

To assess the effect of contrast agent:

- Two chorionic arteries located close to the cord insertion and perfusing different areas of a single placenta were selected.
- One chorionic artery, and therefore placental segment, was perfused with barium sulphate mixed with gelatin (20.8g barium-sulphate (E-Z-Paque, Bracco UK Limited), 50ml 0.9% NaCl and 2.5g gelatine).
- The second chorionic artery, and therefore placental segment, was perfused with a commercial and proprietary lead-based silicon compound called Microfil (Flow Tech, Carver, MA.).

*Perfusion pressure*

To assess the effect of perfusion pressure:

- The placenta was cannulated as described above.
- One chorionic artery, and therefore placental segment, was perfused with Microfil using manual pressure, by gently manually infusing through a 20ml syringe with no quantification of perfusion pressure, but whilst observing for vascular fill and evidence of vascular dilation.
- The second chorionic artery, and therefore placental segment, was perfused with Microfil, using a gravity based perfusion system continuously perfusing the vasculature at a pressure of 60mmHg, physiologically relevant to fetal life[2,3,4].

*Cannulation location*

To assess the effect of cannulation location:

- The umbilical artery of the placenta was cannulated, and the whole placenta perfused with controlled 60mmHg pressure (as described above), perfusing the whole placenta due to Hyrtl’s anastomosis.
- The results were compared with the controlled pressure chorionic artery perfusion of placental segments as described in the previous section.

*Arterial or Venous Cannulation*

To assess the effect of perfusion vessel:

- The umbilical vein of the placenta was cannulated, and the whole placenta perfused with controlled 60mmHg pressure.
- The results were compared with the controlled pressure umbilical artery perfusion of the whole placenta as described in the previous section.

## Placental Histological Analysis

Once fixed, a 10μm full thickness section was cut from each block and stained with hematoxylin and eosin (H&E). Preliminary work showed that the Microfil often fell out of vessels with 3μm and 5μm slide thickness. 10μm was therefore a balance between keeping as much Microfil intravascularly as possible for analysis of vascular fill, and keeping a slide thickness that could be histologically assessed. For each slide, 6 micrographs at x100 magnification were taken, three close to the chorionic plate, and three close to the basal plate, using the template shown in figure 1 as a guide.

1

2

4

6

5

3

Basal Plate

Chorionic Plate

Figure 1: Sampling method for histology.

Micrographs should be taken of each full thickness placental slide using the schematic above, with each numbered box demonstrating the position of a photomicrograph.

Each micrograph was visually inspected for extra-vascular contrast leak and vascular fill. Any slides with no contrast agent were excluded from further analysis. Micrographs were loaded into FIJI (ImageJ Version 2.0.0-rc-54/1.51f)[5], and the scale set. For tissue perfused with barium sulphate the perfused vessels, un-perfused vessels and leaked contrast agent were manually segmented, measuring the area of each. For tissue perfused with Microfil perfused vessels, un-perfused vessels and villous tissue were automatically segmented using the *Trainable Weka Segmentation* plugin in FIJI (Version 3.1.2)[6] and the area of each was automatically measured using the *Analyse Particles* tool. Vascular fill was calculated for each micrograph as shown in equation one.

$$Vascular Fill \left( \% \right)=\left( \frac{Total Perfused Vessel Area}{Total Perfused Vessel Area+Total Unperfused Vessel Area} \right) \times100$$

Equation 1

REFERENCES

[1] A.C. Langheinrich, S. Vormann, J. Seidenstucker, M. Kampschulte, R.M. Bohle, J. Wienhard, M. Zygmunt. Quantitative 3D micro-CT imaging of the human feto-placental vasculature in intrauterine growth restriction. Placenta 29, (2008) 937–41.

[2] P.C. Struijk, V.J. Matthews, T. Loupas, P.A. Stewart, E.B. Clark, E.A.P. Steegers, J.W. Wladimiroff. Blood pressure estimation in the human fetal descending aorta. Ultrasound Obstet. Gynecol. 32, (2008), 673–81.

# [3] B. Castle, I.Z. Mackenzie. *In vivo* observations on intravascular blood pressure in the fetus during mid-pregnancy, in: P. Rolfe Fetal Physiological Measurements. Elsevier, 1986. Pp. 65-69.

[4] P. Johnson, D.J. Maxwell, M.J. Tynan, L.D. Allan. Intracardiac pressures in the human fetus. Heart, 84, (2000) 59–63.

[5] J. Schindelin, C.T. Rueden, M.C. Hiner, K.W. Eliceiri. The ImageJ ecosystem: An open platform for biomedical image analysis. Mol. Reprod. Dev*.* 82, (2015), 518–529.

[6] I. Arganda-Carreras, V. Kaynig, C. Rueden, K.W. Eliceiri, J. Schindelin, A. Cardona, H.S. Seugn. Trainable Weka Segmentation: a machine learning tool for microscopy pixel classification. Bioinformatics (2017)
